# MicroRNA-582-5p Contributes to the Maintenance of Neural Stem Cells Through Inhibiting Secretory Protein FAM19A1

**DOI:** 10.3389/fncel.2022.866020

**Published:** 2022-05-24

**Authors:** Yu-Fei Zhang, Xin-Xin Li, Xiu-Li Cao, Chen-Chen Ji, Xiang-Yu Gao, Dan Gao, Hua Han, Fei Yu, Min-Hua Zheng

**Affiliations:** ^1^State Key Laboratory of Crop Stress Biology for Arid Areas, College of Life Sciences, Northwest A&F University, Yangling, China; ^2^Department of Biochemistry and Molecular Biology, Fourth Military Medical University, Xi’an, China; ^3^Xi’ an Key Laboratory of Stem Cell and Regenerative Medicine, Institute of Medical Research, Northwestern Polytechnical University, Xi’an, China; ^4^Department of Medical Genetics and Developmental Biology, Fourth Military Medical University, Xi’an, China

**Keywords:** neural stem cells, Notch signaling, stemness, miR-582-5p, FAM19A1

## Abstract

Epigenetic regulations on the maintenance of neural stem cells (NSCs) are complicated and far from been fully understood. Our previous findings have shown that after blocking Notch signaling in NSCs *in vivo*, the stemness of NSCs decreases, accompanied by the downregulated expression of miR-582-5p. In the current study, we further investigated the function and mechanism of miR-582-5p in the maintenance of NSCs *in vitro* and *in vivo*. After transfecting a mimic of miR-582-5p, the formation of neurospheres and proliferation of NSCs and intermediate progenitor cells (NS/PCs) were enhanced, and the expression of stemness markers such as Sox2, Nestin, and Pax6 also increased. The results were reversed after transfection of an inhibitor of miR-582-5p. We further generated miR-582 knock-out (KO) mice to investigate its function *in vivo*, and we found that the number of NSCs in the subventricular zone (SVZ) region decreased and the number of neuroblasts increased in miR-582 deficient mice, indicating reduced stemness and enhanced neurogenesis of NSCs. Moreover, RNA-sequencing and molecular biological analysis revealed that miR-582-5p regulates the stemness and proliferation of NSCs by inhibiting secretory protein FAM19A1. In summary, our research uncovered a new epigenetic mechanism that regulates the maintenance of NSCs, therefore providing novel targets to amplify NSCs *in vitro* and to promote neurogenesis *in vivo* during brain pathology and aging.

## Introduction

Neural stem cells (NSCs) are a class of cells with abilities to self-renew and give rise to different neural lineages, such as neurons, astrocytes, and oligodendrocytes (Kriegstein and Alvarez-Buylla, 2009; Hoeck et al., 2010; Imayoshi et al., 2013; Rolando et al., 2016). During embryonic neurogenesis, NSCs divide symmetrically to expand the stem cell population, and divide asymmetrically to produce differentiated progeny cells (Higginbotham et al., 2013; Obernier et al., 2018). Neural progenitors and differentiated neural cells migrate from the germinal zones guided by radial glia cells (RGCs) to reach the mantle layers, and form the cerebral cortex (Hansen et al., 2010; Franco et al., 2012; Kwan et al., 2012). In the adult mammalian brain, on the other hand, NSCs are mainly located in the subventricular zone (SVZ) of the lateral ventricles and the subgranular zone (SGZ) of the hippocampal dentate gyrus (Bond et al., 2015). In the SVZ, adult NSCs (type-B cells) can produce transient amplifying progenitors (TAPs, type-C cells), which then become neuroblasts (type-A cells) after a few divisions (Kokovay et al., 2010). Similarly, in the SGZ, a population of radial glia-like NSCs produce non-radial progenitors, which generate neuroblasts and subsequently differentiate into granule neurons (Kempermann et al., 2015; Sun et al., 2015). During these processes, the maintenance and activation of NSCs and the process of neurogenesis are tightly regulated by various signaling pathways and factors (Urbán et al., 2019; Cao et al., 2021).

MicroRNAs (miRNAs) are evolutionarily conserved, non-coding regulatory RNAs consisting of 20-22 nucleotides. miRNAs can regulate post-transcriptional gene expression by targeting mRNA to inhibit translation or trigger degradation (Pasquinelli, 2012; Grigelioniene et al., 2019), participating in many cellular processes such as development, proliferation, and differentiation, etc. (Song et al., 2013; Shenoy and Blelloch, 2014). To date, many miRNAs have been identified that are specifically or abundantly expressed in mammalian brains (Delaloy et al., 2010; Ponomarev et al., 2011; Piwecka et al., 2017). The temporal and spatial expression characteristics of miRNAs indicate their important roles in the self-renewal, proliferation and differentiation of NSCs (Ji et al., 2013). Notch signaling has been reported to play critical roles in the maintenance, proliferation, and differentiation of NSCs in both developing and adult brains (Aguirre et al., 2010). In our previous work, we have found that the expressions of a group of miRNAs including miR-342-5p and miR-582-5p were changed after Notch signaling blockade in NSCs. Our study has shown that miR-342-5p could promote the transition from NSCs into intermediate neural progenitors (INPs) by targeting GFAP (Gao et al., 2017). As about the miR-582-5p, previous studies have found that miR-582-5p can regulate the proliferation and apoptosis of cancer cells (Zhang et al., 2015; Wang et al., 2021), and affect the progression and metastasis of tumors (Uchino et al., 2013; Chen et al., 2021). Our recent results have revealed that miR-582-5p is involved in early B lymphocyte development by regulating pre-B cell proliferation (Li et al., 2022). However, the function of miR-582-5p in NSCs and neurogenesis has not yet been revealed.

FAM19A1 is a chemokine-like secreted protein, and a member of family with sequence similarity 19 (FAM19). It is highly conserved in vertebrates, and specifically expressed in the central nervous system (CNS) (Tang et al., 2004). It has been suggested that FAM19A1 may play important physiological roles in neurodevelopment and brain functions. Recent studies indicate that FAM19A1 acts as a ligand to bind to the N-terminal domain of the G protein-coupled receptor (GPCR) 1 and activates the Rho-related protein kinase signaling pathway, thereby suppressing the proliferation of NSCs and promoting neuronal differentiation (Zheng et al., 2018). Besides, FAM19A1 KO mice have altered food intake patterns, and become hyperactive with reduced anxiety and reduced sensitivity to pain (Lei et al., 2019). In this study, we demonstrate that miR-582-5p could maintain NSCs *in vivo* and *in vitro*, and promote NSC stemness and proliferation by inhibiting the secreted protein FAM19A1.

## Materials and Methods

### Mice

Using the CRISPR/Cas9 method, miR-582 gene was knocked out in the intron between exons 3 and 4 of the host gene Pde4d. Commercial services for the construction of miR-582 conventional knockout mice were provided by Beijing Biocytogen Co., Ltd. (Beijing, China). The sequences of sgRNAs were 5′-CGTGCAGAGGACATATACACAGG for 5′ guide, and 5′-TGGTACACGGGATTAAGCTCAGG for 3′ guide. This targeting procedure generated 4 founders with about 1,000 bp deletions containing miR-582 gene. One of them (#1,034, indicating a 1,034 bp long sequence deleted) was used as a conventional miR-582 knockout model after confirming the absent expression of miR-582-5p and undisturbed expression of host gene Pde4d (referred to Supplementary Figures 4A,B). For genotyping, tail DNA was extracted from juvenile mice, and subjected to PCR using primers miR582-5′WT-F (5′-TACACGGTAAAATCAGCTACGCGACA) and miR582-5′WT-R (5′-CATGGCCTCTGGCTGAGAGAAACTT) which generate a 414 bp band (wild type allele), and using primers miR582-5′WT-F (5′-TACACGGTAAAATCAGCTACGCGACA) and miR582-3′Mut-R (5′- CCCTCCTGCTTCTACCTCTTGGGAT) which generate a 298 bp band (mutant allele) (Supplementary Figure 4C). Mice were maintained on the C57BL/6 background and in a 12-h light and 12-h dark cycle in specific pathogen-free (SPF) facility. The mice grew grossly normal and lived a normal lifespan. All animal experiments were reviewed and approved by the Animal Experiment Administration Committee of the Fourth Military Medical University to ensure ethnical and humane treatment of animals.

### NS/PCs Culture

Embryonic mouse NS/PCs were isolated from the ganglionic eminences (GE) of E14.5 fetal brain (Chojnacki and Weiss, 2008), and cultured in the proliferation medium at a density of 2 × 10^5^ cells/ml. The proliferation medium contains Neurobasal (Gibco: 21103049, Grand Island, NY, United States) supplemented with 2% B27 supplement (Gibco: 12587010), 1% N2 supplement (Gibco: 17502048), 20 ng/mL recombinant human basic fibroblast growth factor (bFGF, Peprotech: AF-100-18B, Rocky Hill, NJ, United States), 20 ng/mL recombinant human epidermal growth factor (EGF, Peprotech: AF-100-15), 2 mM L-glutamine (Gibco: 25030081), 1% penicillin/streptomycin (Gibco: 15140163). Cells were cultured at 37°C with 5% CO_2_. Half-volume of the medium was replaced with fresh medium on the 3rd day of the culture, and the number of neurospheres was counted on the 6th day after the setting of the culture. During cell passaging, spheres were collected by centrifugation at 100 g × 5 min, then digested with Accutase (Gibco: A1110501) for 5 min at 37°C to disperse into single cells.

### Notch Signal Blocking Assay

NSCs were cultured in the proliferation medium supplemented with the gamma-secretase inhibitor (GSI, Abcam: ab142164, Cambridge, MA, United States) at the concentration of 75 μM for 6 days, with adding the same volume of dimethyl sulfoxide (DMSO, Sigma-Aldrich: D4540, St. Louis, MO, United States) as a control. Half-volume of the medium was replaced with fresh medium containing GSI or DMSO on the 3rd day of the culture. Neurospheres were then collected on the 6th day, total RNA was extracted for further analyses.

### Cell Transfection

Mature neurospheres were collected by centrifugation at 100 g × 5 min, digested and dispersed by Accutase for 5 min at 37°C, and then seeded into a six well cell culture plate in Opti-MEM (Gibco: 31985070) at a density of 5 × 10^5^ cells/ml. NSCs were then transfected with the miR-582-5p mimic (5′-auacaguuguucaaccaguuac), inhibitor (5′-guaacugguugaacaacuguau), or their scrambled control oligonucleotides (Ribobio: miR1N0000001-1-5 as NC-mimic, miR2N0000001-1-5 as NC-inhibitor, Guangzhou, Guangdong, China), respectively. For small interfering RNA (siRNA) experiments, NSCs were transfected with si-FAM19A1 (5′-aauucguguagucuuaauc), or the scrambled control oligonucleotides (Ribobio: siN0000001-1-5). The transfection concentration of mimic, NC-mimic, si-FAM19A1 and NC-siRNA were 50 nM, and the concentration of inhibitor and NC-inhibitor were 100 nM according to the manufacturer’s instructions. They were added to the NS/PCs after 20 min of co-incubation with lipofectamine LTX (Invitrogen: A12621, Carlsbad, CA, United States) in Opti-MEM according to the manufacturer’s instructions. Six hours after transfection, proliferation medium was added into each well. Sphere formation assay or 5-ethynyl-2′-deoxyuridine (EdU) labeling was performed 24 h after transfection, and RNA or protein was collected 48 h after transfection. Scrambled control oligonucleotides labeled with 6-carboxy-fluorescein (FAM, Ribobio: siT0000001-1-1) were transfected to monitor the transfection efficiency.

### Sphere Formation Assay

After transfection, NS/PCs were cultured for 24 h, then digested with Accutase for 5 min at 37°C, and collected by centrifugation at 200 g for 5 min. Cells were counted and replated at density of 2 × 10^4^/ml in 24 well plate, 3 experimental replicates of wells per group. Half-volume of the medium was replaced with fresh proliferation medium on the 3rd day of the culture. On the 6th day from replating, neurospheres were observed and photographed under a microscope (Eclipse Ti, Nikon). Images were acquired using a Plan Fluor 4 × NA 0.13 objective, at 2,930 μm × 2,930 μm (with an image resolution of 1.82 μm/px). The diameters of neurospheres were measured by Image-Pro Plus 6.0. Those with diameters longer than 50 μm were regarded as clonal neurospheres, and the total numbers of neurospheres per well were counted.

### 5-Ethynyl-2′-Deoxyuridine Labeling

After transfection, NS/PCs were cultured for 24 h, then digested with Accutase, and collected by centrifugation at 200 g for 5 min. Cells were counted and replated onto Poly-L-lysine (PLL, Sigma-Aldrich: P6282)-coated slides at the density of 4 × 10^5^/ml, in a 24 well plate, 3 experimental replicates of wells per group. And EdU was incorporated using a kit (Ribobio: C10310-1) for 24 h, and slides were stained according to the instructions from the supplier and images were taken under a fluorescence microscope (Eclipse Ni, Nikon). The objective lens used were Plan Fluor 10 × NA 0.30 and Plan Fluor 40 × NA 0.60, and the images were collected at 1,200 μm × 1,200 μm (with an image resolution of 0.75 μm/px). Five random and none-overlapping fields were photographed per slide, and the ratio of EdU positive cells in total Hoechst labeled cells were counted and calculated by Image Pro-Plus.

### Real-Time Quantitative PCR Analysis

Embryonic cortex and GE tissues were dissected and collected according to previous literature (Chojnacki and Weiss, 2008), and adult tissues of liver, kidney, and cortex were dissected from 8 weeks old C57BL/6 mice. Total RNA from tissue or NS/PCs was extracted with Trizol (Invitrogen: 15596018), and was used to synthesize cDNA with Mir-X miRNA First-Strand Synthesis Kit (TaKaRa: 638313, Tokyo, Japan) for miRNA, and PrimeScript RT Master Mix (TaKaRa: RR036A) for mRNA. Real-time quantitative PCR (RT-qPCR) was performed on the Thermofisher QuantStudio 5 real-time PCR system, and TB Green^®^ Premix Ex Taq™ II (TaKaRa: RR820A) was used for amplification and quantification. The U6 RNA and b-actin mRNA were used as internal controls for miRNA and mRNA analyses, respectively. The primer sequences of RT-qPCR are listed in Table 1.

**TABLE 1:** List of primers.

Name	Forward primer	Reverse primer
miR-582-5p	ATACAGTTGTTCAACCAGTTAC	mRQ 3′ primer
U6	GGATGACACGCAAATTCGTGAAGC	mRQ 3′ primer
Hes1	AAAGACGGCCTCTGAGCAC	GGTGCTTCACAGTCATTTCCA
Hes5	AGTCCCAAGGAGAAAAACCGA	GCTGTGTTTCAGGTAGCTGAC
Pre-miR-582	ACTCTTTGGATACAGTTGTT	TTTGCACCCTTTGGGTTCAG
Pde4d	TTTTGCCAGTGCAATACATGATG	CAGAGCGAGTTCCGAGTTTGT
Sox2	GCGGAGTGGAAACTTTTGTCC	CGGGAAGCGTGTACTTATCCTT
Nestin	TGAAAAGTTCCAGCTGGCTGT	AGTTCTCAGCCTCCAGCAGAGT
Pax6	TACCAGTGTCTACCAGCCAAT	TGCACGAGTATGAGGAGGTCT
CD133	ACTGAGAAATCCCCTACTGAAGT	GGCCTGTTTCGGCTTTCCTT
Glast	ACCAAAAGCAACGGAGAAGAG	GGCATTCCGAAACAGGTAACTC
Vimentin	CGTCCACACGCACCTACAG	GGGGGATGAGGAATAGAGGCT
FAM19A1	GTGTTCCTGTTTACCTGGGAAA	GCTCCATCTCACACCACCATT
Nuggc	TGAAAAACTGGAGTGTCGGAC	GCAAGAAGCCGGTTTATGAGATA
Grifin	CATCGCCTTCCACGTCAAAC	CCAGAGTCAGTGGGAATATGCT
Slc25a48	CTGGAAGACTTTGTGGCAGG	GTTCGCATAGCCGACACCA
Dmrtc1a	GGAACCTCGTAAGGACTTTTCTC	TGCATGTGTGATGGATGAGCA
Zbtb9	ATGGATGCTTCGACTCCTTTG	TTGTGAGCCCTAAGTTCCCTG
Luciferase	GCCTACCGTGGTGTTCGTTT	AGGCAGAGCGACACCTTTAG
β-actin	CATCCGTAAAGACCTCTATGCCAAC	ATGGAGCCACCGATCCACA

### Western Blotting

Total proteins were extracted with RIPA lysis buffer (Beyotime: P0013B, Shanghai, China) added with 1 mM Phenylmethanesulfonyl fluoride (PMSF, Beyotime: ST506), and the protein concentration in the supernatants was determined by enhanced BCA protein detection kit (Beyotime: P0012). Equal amounts of proteins were separated by 12% SDS-PAGE and blotted onto 0.45 μm polyvinylidene fluoride (PVDF) membranes (Millipore: IPVH00010, MA, United States). After blocked with 5% skim milk (Beyotime: P0216) in PBST at room temperature for 2 h, the blots were probed with the following primary antibodies at 4°C for 12 h: anti-Sox2 (1:1,000, Abcam: ab92494), anti-Nestin (1:1,000, BD: 556309, Franklin Lake, NJ, United States), anti-Pax6 (1:1,000, BioLegend: 901301), anti-FAM19A1 (1:1,000, R&D: AF5154, Minneapolis, MN, United States), and anti-β-actin (1:5,000, Proteintech: 66009-1). The secondary antibodies were incubated at room temperature for 2 h: horseradish peroxidase (HRP)-conjugated mouse anti-rabbit IgG (1:5,000, Abcam: ab99697), HRP-conjugated rabbit anti-mouse IgG (1:5,000, Abcam: ab6728), HRP-conjugated rabbit anti-rat IgG (1:5,000, Abcam: ab6734), and HRP-conjugated donkey anti-goat IgG (1:5,000, Abcam: ab6885). All antibodies were diluted with PBST. Images were obtained with GBOX-CHEMI-XX9-E (Syngene, Cambridge, United Kingdom). And image quantification and analysis were performed using ImageJ software.

### Immunofluorescence Staining and Confocal Microscopy

Mice (8 weeks old) were perfused with 40 ml PBS. The brains were carefully removed, coated with O.C.T. Compound (Sakura: 4583, CA, United States), and fast frozen by liquid nitrogen to avoid the formation of crystals, then stored at −80 °C. Frozen sections were cut with 14 μm depth by a cryostat (Leica, CM1950) and mounted on gelatinized slides. The sections were fixed with 4% PFA for 5 min before staining, and then washed with PBS for 3 times. They were incubated in PBS/0.1% Triton X-100 containing 1% BSA for 30 min and then incubated with primary antibodies overnight at 4^°^C. The primary antibodies used for incubation are as follows: anti-GFAP (1:1,000, Abcam: ab53554), anti-Sox2 (1:500, Abcam: ab92494), anti-EGFR (1:500, Abcam: ab52894), anti-Dcx (1:500, Abcam: ab18723), anti-CD24 (1:500, BD: 553146), and anti-Ki67 (1:500, Abcam: ab16667). The secondary antibodies were incubated at room temperature for 2 h, including Alexa Fluor 594 donkey anti-goat (1:1,000, Invitrogen: A32758), Alexa Fluor 488 donkey anti-rabbit (1:1,000, Invitrogen: A21206), and Alexa Fluor 488 donkey anti-rat (1:1,000, Invitrogen: A21208). The immunostained brain sections were visualized and imaged under a fluorescence microscope (Eclipse Ni, Nikon) or a laser scanning confocal microscope (A1R confocal microscope, Nikon). The objective lens used were Plan APO 20 × NA 0.75 and Plan Fluor 40 × NA 0.75, and the images were collected at 630 μm × 630 μm (with an image resolution of 0.62 μm/px). Six pairs of gender-matched animals were measured for each staining. From each pair of mice, 14 μm sections of SVZ (40 sections) were collected, and the one representative section from each five successive sections was chosen for counting and measurement. At least five sections were counted for each measurement. And Image Pro-Plus was used for image quantification and analysis. The striatal side of SVZ was analyzed for GFAP and Sox2, EGFR, DCX and Ki67 staining, and the size of analyzed field was 380 μm × 630 μm (width × height). For CD24 staining, the striatal side, septal side and corpus callosum side of SVZ were all analyzed. For 3D images of confocal imaging, the thickness of the analyzed field was 10 μm, and the Z step size was 1 μm. For quantification of cells positive for any single marker, the number of positive cells was manually counted in the field. For double-immunofluorescence staining, NIS-Elements (Nikon) was used for image acquisition, background correction, and image merging, and then nuclei with overlapping fluorescent signals were counted.

### RNA-Seq Analysis

After transfection of miR-582-5p mimic or the negative control, the total RNA of transfected NS/PCs was extracted according to the manufacturer’s instructions. And 3 biological replicates were carried out for each group. The RNA samples were submitted to the Shanghai Personal Biotechnology Co., Ltd. (Shanghai, China) for transcriptome sequencing using Illumina NovaSeq 6000. The online OmicShare tools^1^ were used for the subsequent bioinformatic analysis such as the Expression analysis, the differentially expressed genes (DEGs) analysis, and the Functional enrichment analysis of DEGs.

### Dual-Luciferase Reporter Assay

For Pde4d promoter reporter construction, the promoter DNA fragment (-2370 bp upstream to 474 bp downstream of the mouse Pde4d transcription start site) were cloned and inserted upstream of the luciferase gene in the pGL3-basic vector (Promega: E1751, Madison, WI, United States). The Pde4d promoter fragment contains two binding sites of RBP-J (5′-GTGGGAA-3′). For FAM19A1 3′UTR reporter construction, the 3′UTR DNA fragment (position 2,103–2,837 bp of mouse FAM19A1 3′ UTR) were cloned and inserted downstream of the luciferase gene in the pMIR-REPORT vector (Gao et al., 2017). The 3′UTR fragment contains a target site of miR-582-5p (5′-ACTGTAA-3′).

HEK293T cells were cultured in DMEM media (Gibco: 11995065) with 10% fetal bovine serum (Gibco: 10099141). Cells were plated at the density of 5 × 10^5^/ml in 48 well plate for 24 h before transient transfection. Cells were transfected with different combinations of reporters and expression vectors (or mimics) by using Lipofectamine 2000 (Invitrogen: 11668019), with a Renilla luciferase vector (phRL-TK, Promega) as an internal control. Cells were harvested 48 h after the transfection, lysed with 1 × passive lysis buffer (Promega: E1910) for 20 min on ice. Then supernatants were collected by centrifugation at 12,000 g × 10 min at 4°C. Luciferase activity of cell extracts was measured by using a dual luciferase reporter assay kit (Promega: E1960) through a Gloma X™ 20/20 Luminometer (Promega: E5311).

### Cell Cycle Analysis by Flow Cytometry

NS/PCs were collected 48 h after transfection. After washed by pre-cooled PBS, cells were filtered through 60 μm Nylon Net Filter (Millipore: NY6000010), and then fixed with 70% alcohol at 4°C for 30 min. Cells were then centrifuged at 200 g for 5 min, washed with pre-cooled PBS, resuspended in 200 μl PBS, and added RNase (working concentration for 100 μg/ml) at 37°C for 30 min. Afterward, Propidium Iodide (PI, working concentration for 50 μg/ml, Sigma-Aldrich P4170) was added, and cells were stained in the dark on ice for 30 min. Cell cycle was detected by flow cytometry (BD FACSCalibur).

### Statistical Analysis

All statistical analyses were performed in Prism 7.0 (GraphPad Software), using Student’s *t*-test and analysis of variance (ANOVA). D’Agostino-Pearson omnibus (K2) was used for normality analysis. Welch’s *t*-test was used when the variances were uneven. Dunnett’s test was used for the *post hoc* analysis in one-way ANOVA and two-way ANOVA. Statistical significance was defined as *p* < 0.05, *p* < 0.01, or *p* < 0.001 as specified in the figure legend. Values are from 3 to 6 independent experiments with multiple replicates each and reported as mean ± SEM.

## Results

### miR-582-5p Is Expressed in Neural Stem Cells and Regulated by Notch Signaling

**FIGURE 1:**
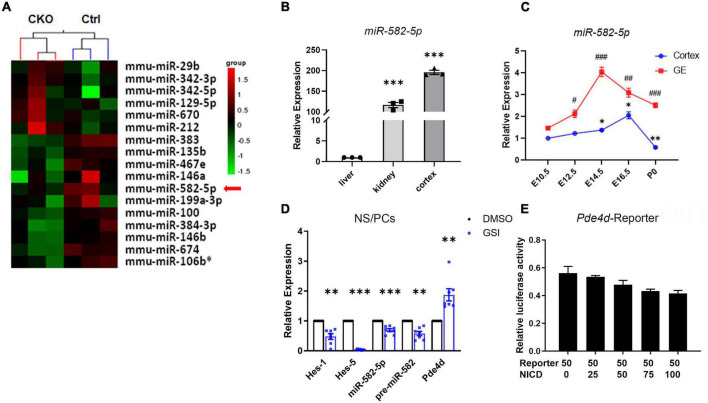
miR-582-5p is expressed in the brain and is positively regulated by Notch signaling. **(A)** microRNA array showed that the expression of miR-582-5p in NSCs was down-regulated in RBP-J CKO mice compared with Control mice (*p* < 0.05). **(B)** RT-qPCR analysis of miR-582-5p in liver, kidney, and cortex of adult C57BL/6 mice, *n* = 3. One-way ANOVA with Dunnett’s test. **(C)** RT-qPCR analysis of miR-582-5p in cortex (blue) and in ganglionic eminences (GE, red) at E10.5, E12.5, E14.5, E16.5, and P0 of C57BL/6 mice, *n* = 3. One-way ANOVA with Dunnett’s test. **(D)** RT-qPCR analysis of Notch downstream genes Hes1 and Hes5, miR-582-5p, precursor of miR-582-5p, and host gene Pde4d in control and GSI-treated NSCs, *n* = 7. **(E)** The relative luciferase activity of the Pde4d promoter reporter with the increase of NICD concentration, *n* = 3. One-way ANOVA with Dunnett’s test. For statistical analysis, *^#^*p* < 0.05, **^##^*p* < 0.01, ***^###^*p* < 0.001, compared with the control, respectively.

Our previous research (Gao et al., 2017) showed that in Nestin-cre; RBP-J-floxed conditional knockout (cKO) mice, in which the canonical Notch signaling is specifically blocked in NSCs, the expression of miR-582-5p decreased (Figure 1A), indicating that it may participate in regulating NSCs. Therefore, we first examined the expression of miR-582-5p in neural tissues. In adult C57BL/6 mice, miR-582-5p is highly expressed in brain cortex compared with liver and kidney (Figure 1B). During the embryonic development of the brain, the expression of miR-582-5p in the ganglionic eminences (GE) reached its peak at embryonic day (E) 14.5, and in the cortex at E16.5 (Figure 1C). For further research, NS/PCs were isolated from the GE of fetal mouse brain at E14.5 by *in vitro* cultivating to form neurospheres. The results of real-time quantitative PCR (RT-qPCR) showed that miR-582-5p is expressed in NS/PCs (Figure 1D), and after Notch signaling was blocked in NS/PCs by a gamma-secretase inhibitor (GSI), the expression of miR-582-5p and its precursor miR-582 were decreased, together with reduced expression of typical Notch downstream genes such as Hes1 and Hes5, indicating that Notch signaling positively regulates the expression of miR-582-5p (Figure 1D). The miR-582-5p gene locates within the third intron of Pde4d. However, dual-luciferase reporter assay showed that Notch signaling did not directly regulate the Pde4d promoter activity (Figure 1E). These results indicate that miR-582-5p is expressed in NSCs and is indirectly regulated by Notch signaling.

### miR-582-5p Promotes the Neurosphere Formation and Proliferation of NS/PCs *in vitro*

**FIGURE 2:**
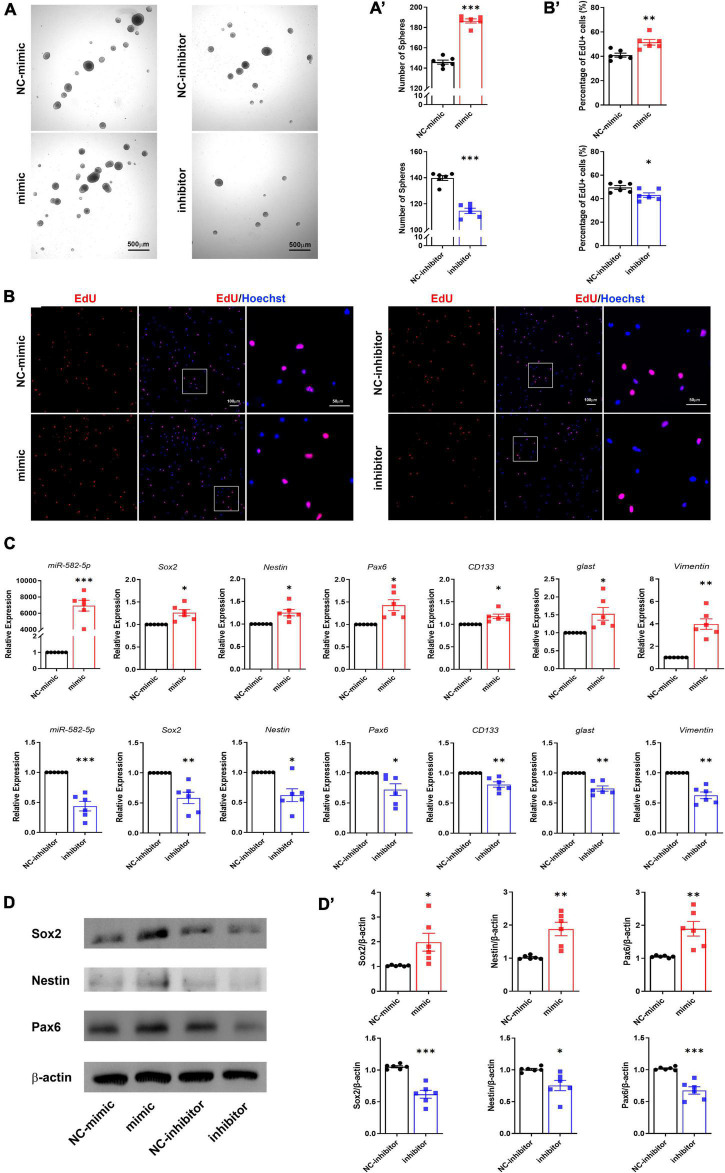
miR-582-5p promotes neurosphere formation and proliferation of NS/PCs *in vitro*. **(A)** Bright field microscopy of neurosphere assay in mimic/inhibitor group and negative control (NC) group, *n* = 6, Scale bar = 500 μm. **(A’)** Corresponding quantifications and statistics of neurosphere number. **(B)** EdU labeling of mimic/inhibitor group and NC group, *n* = 6, Scale bar = 100 μm, 50 μm. **(B’)** Corresponding quantifications and statistics of EdU labeling. **(C)** RT-qPCR of miR-582-5p and stemness markers Sox2, Nestin, Pax6, CD133, glast, and Vimentin in mimic/inhibitor group and NC group, *n* = 6. **(D)** Western blotting of Sox2, Nestin, and Pax6 in mimic/inhibitor group and NC group, *n* = 6. **(D’)** Corresponding quantifications and statistics of **(D)**. For statistical analysis, *p < 0.05, **p < 0.01, ***p < 0.001, compared with the control, respectively.

To further determine the function of miR-582-5p in the maintenance of NSCs, NS/PCs dissociated from cultured neurospheres were identified by Sox2 immunostaining and transfected with Scrambled control oligonucleotides labeled with FAM to monitor the transfection efficiency (Supplementary Figure 1). Then NS/PCs were transfected with chemically synthesized mimic or inhibitor of miR-582-5p and the corresponding negative control, respectively. After transfection of miR-582-5p mimic, RT-qPCR verified that the expression of miR-582-5p was significantly increased (Figure 2C), and the number of neurospheres was increased compared with that of the control (Figures 2A,A’), indicating the enhanced neurosphere formation after the transfection of the miR-582-5p mimic. On opposite, the level of miR-582-5p was decreased after the miR-582-5p inhibitor transfection as shown by RT-qPCR (Figure 2C), and there were fewer neurospheres in the inhibitor transfection group compared with that of the control (Figures 2A,A’), verifying miR-582-5p’s function in promoting neurosphere formation. Besides, the diameter of spheres in the inhibitor transfection group was shorter than that of the control group (Supplementary Figure 2A). Moreover, the percentage of 5-ethynyl-2′-deoxyuridine (EdU) positive cells was higher in the mimic transfection group and was lower in the inhibitor transfection group, compared with that of the control, respectively (Figures 2B,B’). In addition, the cell cycle analysis on NS/PCs by Flow Cytometry revealed the accumulation of G2-M and S phase cells and reduction of G1-G0 phase cells after miR-582-5p overexpression (Supplementary Figure 3). All these results indicate that miR-582-5p also promotes the proliferation of NS/PCs.

RT-qPCR results showed that the stemness markers, such as Sox2, Nestin, Pax6, CD133, glast and Vimentin were all up-regulated in the miR-582-5p mimic transfection group compared with the control group (Figure 2C). Western blotting analysis also proved increased expression of Sox2, Nestin and Pax6 at the protein level (Figures 2D,D’). Meanwhile, the expression of stemness markers in the miR-582-5p inhibitor-transfected group were down-regulated both at mRNA and protein levels (Figures 2C,D,D’). The above results further confirmed that miR-582-5p promotes the stemness and proliferation of NSCs.

### The Stemness of Neural Stem Cells Declines in Subventricular Zone of miR-582 Knock-Out Mice

**FIGURE 3:**
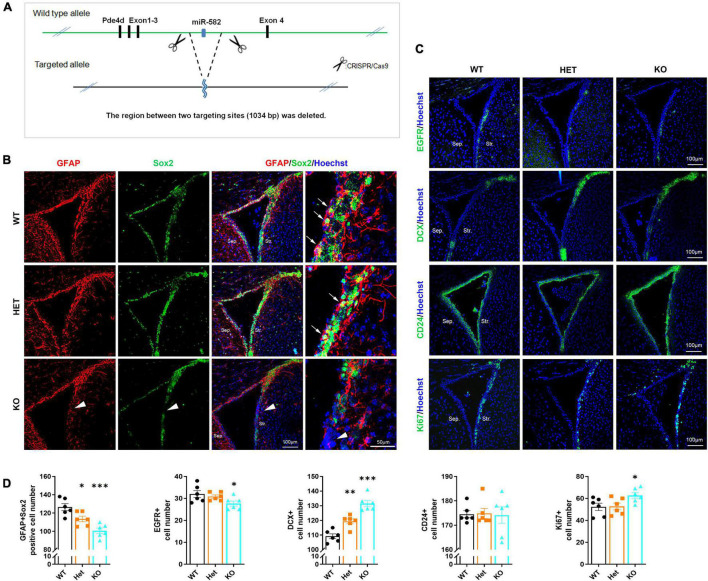
Reduced NSCs and increased neuroblasts in miR-582 KO mice. **(A)** Diagram of miR-582 KO mice construction. **(B)** Immunofluorescence analysis of GFAP^+^Sox2^+^ NSCs, *n* = 6, Scale bar = 100, 50 μm. One-way ANOVA with Dunnett’s test. **(C)** Immunofluorescence analysis of EGFR^+^ TAPs, DCX^+^ neuroblasts, CD24^+^ ependymal cells, and Ki67^+^ cells. *n* = 6, Scale bar = 100 mm. One-way ANOVA with Dunnett’s test. **(D)** Corresponding quantifications and statistics of immunostaining positive cells per field in **(B,C)**. For statistical analysis, *p < 0.05, **p < 0.01, ***p < 0.001, compared with the WT group. Sep., Septal; Str., Striatal. Arrows indicate GFAP^+^Sox2^+^ NSCs, and arrowheads indicate the decrease of GFAP^+^Sox2^+^ NSCs in miR-582 KO SVZ sections.

To further observe the role of miR-582-5p in NSCs *in vivo*, we deleted miR-582 gene in the intron between exons 3 and 4 of the host gene Pde4d through the CRISPR/Cas9 system to construct miR-582 knockout (KO) mice (Figure 3A). We found that expression of miR-582-5p was absent in the miR-582 KO brain (Supplementary Figure 4A), without disturbance of expression of its host gene Pde4d (Supplementary Figure 4B). The viability and gross brain morphology of wild type control (WT), heterozygous (Het), and homozygous KO mice were comparable (Supplementary Figure 5), excluding severe defects at embryonic developmental stages. Therefore, we focused on NSCs in adult miR-582 KO mice aged 8 weeks.

NSCs, TAPs and neuroblasts in SVZ were detected by immunofluorescence staining of frozen brain sections of WT, Het, and KO mice. In the SVZ of KO group, the number of GFAP^+^Sox2^+^ NSCs (arrows in Figure 3B) and EGFR^+^ TAPs (Figure 3C) were decreased (for GFAP^+^Sox2^+^ NSCs, arrowheads in Figure 3B) compared with those of WT (Figures 3B–D), indicating reduced NSCs and TAPs in SVZ. And the DCX^+^ neuroblasts were increased compared with that of the WT group (Figures 3C,D). Moreover, the number of Ki67^+^ cells in the SVZ of KO group was more than that of the WT group (Figures 3C,D). The results of the Het group were between the KO and WT groups. In addition, there was no significant difference in the number of CD24^+^ ependymal cells among the three groups (Figures 3C,D). These results implied that in the brain of miR-582 KO mice, type-B NSC pool reduced, and type-A neuroblasts expanded, suggesting an enhanced neurogenesis process. Therefore, the stemness of NSCs decreased in SVZ of adult miR-582 KO mice.

### miR-582-5p Down-Regulates the Expression of FAM19A1

**FIGURE 4:**
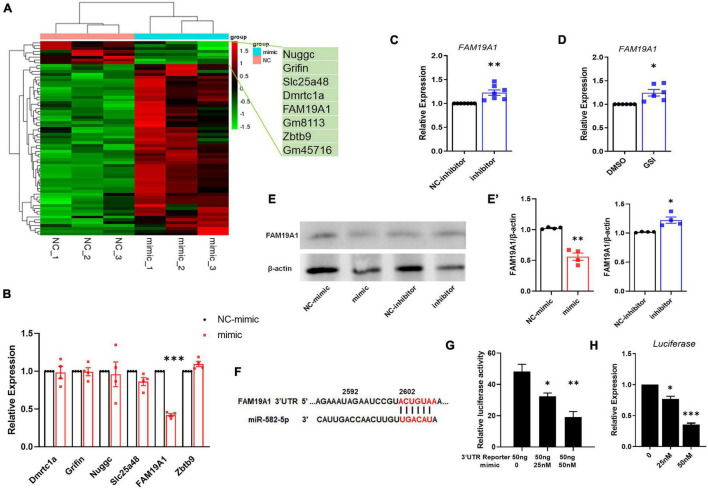
miR-582-5p down-regulates the expression of FAM19A1. **(A)** RNA-seq of miR-582-5p mimic and NC-mimic transfected NSCs, *n* = 3. **(B)** The mRNA expression of potential target genes in the mimic and NC-mimic group, with FAM19A1 down-regulated, *n* = 4. **(C)** RT-qPCR analysis of FAM19A1 in the inhibitor and NC-inhibitor group, *n* = 7. **(D)** RT-qPCR analysis of FAM19A1 in the GSI and control DMSO group, *n* = 6. **(E,E’)** Western blotting and statistics of FAM19A1 in the mimic/inhibitor and control group, *n* = 4. **(F)** The sequence of the FAM19A1 3′UTR and the binding site of miR-582-5p (red font). **(G)** The relative luciferase activity of the FAM19A1 3′UTR reporter with the increase of miR-582-5p concentration, *n* = 3. One-way ANOVA with Dunnett’s test. **(H)** The expression of firefly luciferase mRNA with the increase of miR-582-5p concentration, *n* = 4. One-way ANOVA with Dunnett’s test. For statistical analysis, *p < 0.05, **p < 0.01, ***p < 0.001, compared with the control, respectively.

To explore the mechanism of miR-582-5p affecting NSC stemness, we transfected miR-582-5p mimics in cultured neurospheres from E14.5 embryos, and performed RNA sequencing. The results showed that 60 genes were up-regulated and 8 genes were down-regulated in neurospheres transfected with the miR-582-5p mimic (Figure 4A). Among the down-regulated genes, 6 potential target genes were tested for further study, with FAM19A1 most significantly down-regulated in the mimic group (Figure 4B). On the contrary, the expression of FAM19A1 was up-regulated in neurospheres transfected with the miR-582-5p inhibitor (Figure 4C). Moreover, since Notch signaling positively regulates the expression of miR-582-5p, we also tested the expression of FAM19A1 in neurospheres treated with GSI, and we found that FAM19A1 expression was also up-regulated (Figure 4D). The protein level of FAM19A1 was down-regulated in the miR-582-5p mimic-transfected group and up-regulated in the inhibitor-transfected group (Figures 4E,E’), in correspondence with the mRNA level. To further confirm the physical interaction and post-transcriptional regulation on the 3′UTR fragment of FAM19A1 by miR-582-5p, we used genome DNA PCR to clone the mouse FAM19A1 3′UTR containing putative binding site of miR-582-5p (Figure 4F) and constructed the according luciferase reporter plasmid. As shown in Figure 4G, we used transient transfection to co-introduce luciferase plasmid containing the 3′UTR region of FAM19A1 with miR-582-5p mimic or NC oligonucleotides into HEK293T cells. The result of dual luciferase reporter assay showed that the normalized luciferase activity gradually decreased with the increase of the concentration of the miR-582-5p mimic (Figure 4G), indicating that the 3′UTR region of FAM19A1 could specifically bind and be inhibited by miR-582-5p. Besides, the expression of firefly luciferase mRNA decreased with increasing concentration of miR-582-5p mimic (Figure 4H), indicating miR-582-5p inhibited FAM19A1 by regulating its mRNA stability. Collectively, these results demonstrated that FAM19A1 is a direct target of miR-582-5p.

### Inhibition of FAM19A1 Enhances the Stemness of Neural Stem Cells

**FIGURE 5:**
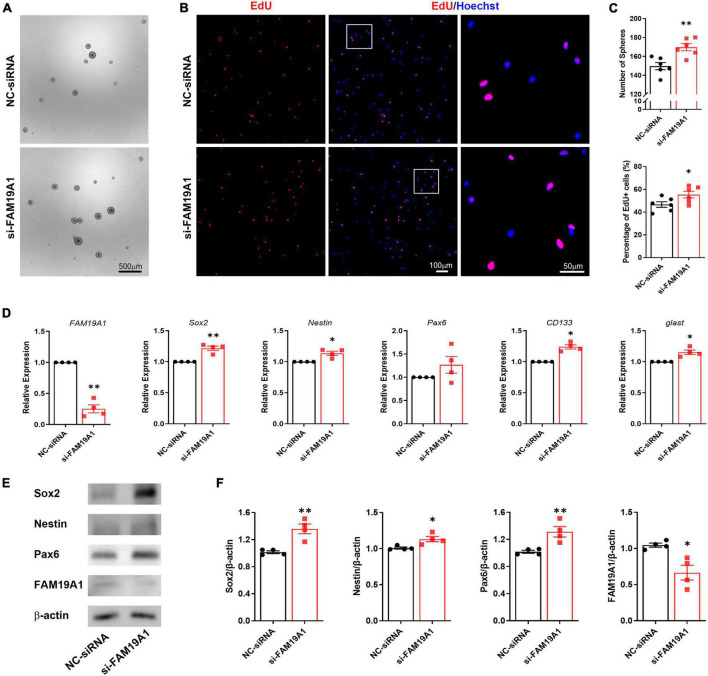
The inhibition of FAM19A1 promotes stemness and proliferation of NSCs. **(A)** Bright field microscopy of neurospheres in si-FAM19A1 and control group, *n* = 6, Scale bar = 500 μm. **(B)** EdU labeling in si-FAM19A1 and control group, *n* = 6, Scale bar = 100, 50 μm. **(C)** Corresponding quantifications and statistics of **(A,B)**. **(D)** RT-qPCR of FAM19A1 and stemness markers Sox2, Nestin, Pax6, CD133, and glast, *n* = 4. **(E)** Western blotting analysis of Sox2, Nestin, and Pax6, *n* = 4. **(F)** Corresponding quantifications and statistics of **(E)**. For statistical analysis, *p < 0.05, **p < 0.01, compared with the NC-siRNA group.

To further explore the function of FAM19A1 in NSCs, a synthesized si-FAM19A1 was transfected into primary cultured NS/PCs, with si-Scramble RNA as a control (NC-siRNA). The number of spheres in si-FAM19A1-transfected group was increased compared with that of control group (Figures 5A,C). Besides, the diameter of spheres in si-FAM19A1-transfected group was longer than that of control group (Supplementary Figure 2B). The percentage of EdU positive cells was also increased after si-FAM19A1 transfection (Figures 5B,C). Moreover, the results of RT-qPCR showed that with FAM19A1 expression in the si-FAM19A1-transfected group significantly decreased, the mRNA expression of stemness markers were up-regulated (Figure 5D). Further Western blotting analysis also indicated increased protein expression of Sox2, Nestin and Pax6 (Figures 5E,F). The above results demonstrated that inhibition of FAM19A1 promotes the stemness and proliferation of NSCs.

### Inhibition of FAM19A1 Could Rescue the Weakened Stemness and Proliferation of Neural Stem Cells Caused by miR-582-5p Inhibition

**FIGURE 6:**
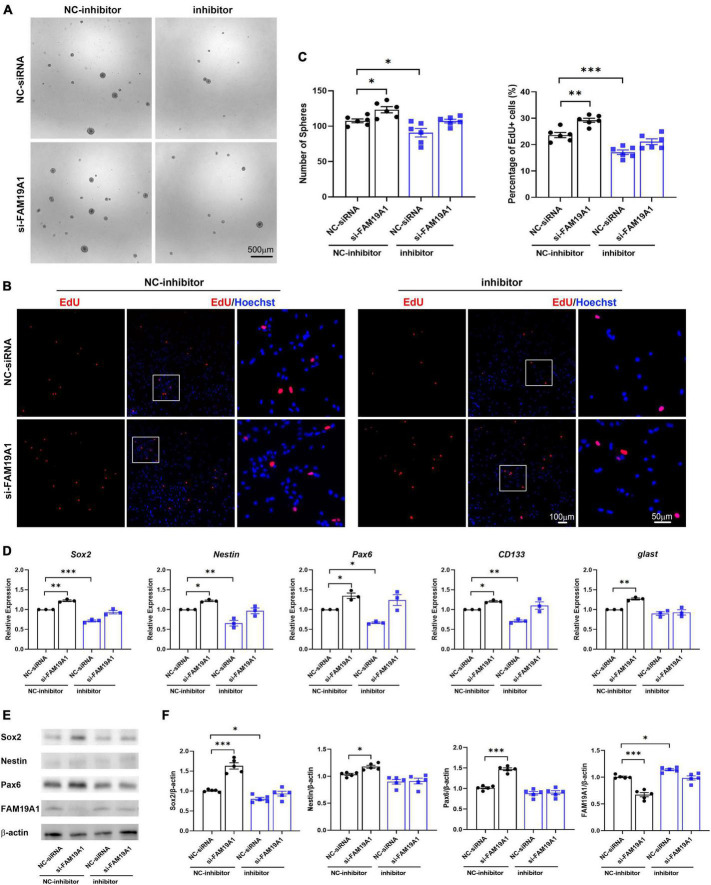
si-FAM19A1 could rescue weakened stemness and proliferation of NSCs caused by miR-582-5p inhibitor. **(A)** Bright field microscopy of neurospheres in the NC-inhibitor and NC-siRNA co-transfection group, the NC-inhibitor and si-FAM19A1 group, the miR-582-5p inhibitor and NC-siRNA group, and the miR-582-5p inhibitor and si-FAM19A1 group, *n* = 6, Scale bar = 500 μm. **(B)** EdU labeling of the four groups, *n* = 6, Scale bar = 100, 50 μm. **(C)** Corresponding quantifications and statistics of A and B. Two-way ANOVA with Dunnett’s test. **(D)** RT-qPCR of stemness markers Sox2, Nestin, Pax6, CD133, and glast, *n* = 3. Two-way ANOVA with Dunnett’s test. **(E)** Western blotting analysis of Sox2, Nestin, and Pax6, *n* = 5. **(F)** Corresponding quantifications and statistics of E. Two-way ANOVA with Dunnett’s test. For statistical analysis, *p < 0.05, **p < 0.01, ***p < 0.001, compared with the control, respectively.

In order to study whether miR-582-5p regulates NSCs through FAM19A1, si-FAM19A1 was co-transfected with the miR-582-5p inhibitor in cultured NS/PCs. The number of neurospheres in the miR-582-5p inhibitor and NC-siRNA co-transfection group was decreased compared with the NC-inhibitor and NC-siRNA co-transfection group. However, the miR-582-5p inhibitor and si-FAM19A1 co-transfection could rescue the decreased number of neurospheres (Figures 6A,C). Besides, the percentage of EdU positive cells in the miR-582-5p inhibitor and NC-siRNA co-transfection group was decreased compared with the NC-inhibitor and NC-siRNA co-transfection group, and it was recovered in the miR-582-5p inhibitor and si-FAM19A1 co-transfection group (Figures 6B,C). The two-way ANOVA analysis showed significant effects of inhibitor or si-FAM19A1 transfection in neurosphere formation assay (*p* = 0.0008 for inhibitor, *p* = 0.0010 for si-FAM19A1) and EdU labeling assay (*p* < 0.0001 for both), while the interaction between inhibitor transfection and si-FAM19A1 transfection was not significant (*p* = 0.9212 for sphere formation assay, *p* = 0.3945 for EdU labeling assay).

Moreover, the results of RT-qPCR showed that expression of stemness markers decreased in the miR-582-5p inhibitor and NC-siRNA co-transfection group compared with the control group, and recovered in the miR-582-5p inhibitor and si-FAM19A1 co-transfection group (Figure 6D). Western blotting analysis confirmed the restoration of Sox2 at the protein level in the miR-582-5p inhibitor and si-FAM19A1 co-transfection group, compared with the miR-582-5p inhibitor and NC-siRNA co-transfection group (Figures 6E,F). The two-way ANOVA analysis showed significant effects of inhibitor or si-FAM19A1 transfection in the mRNA expression (*p* < 0.05 for all) and the protein expression (*p* < 0.05 for all except Nestin) of stemness markers. The interaction between inhibitor transfection and si-FAM19A1 transfection was not significant in RT-qPCR analysis except glast (*p* = 0.9596, 0.4004, 0.1894, 0.0995, 0.0236, for the mRNA expression of Sox2, Nestin, Pax6, CD133, glast, respectively), and was significant in Western blotting analysis except Nestin (*p* = 0.0003, 0.1067, 0.0001, 0.0089, for the protein expression of Sox2, Nestin, Pax6, FAM19A1, respectively). The above results demonstrated that inhibition of FAM19A1 could rescue the weakened stemness and proliferation of NSCs caused by miR-582-5p inhibition.

## Discussion

microRNA-582-5p has been found to be relatively abundant in neural tissues and was down-regulated in NSCs with Notch signal blocked. In this study, we further demonstrated that Notch signaling indirectly regulates the expression of miR-582-5p. Since Notch signaling plays pivotal roles in maintaining NSC stemness (Imayoshi et al., 2010; Matsumoto et al., 2011), miR-582-5p is like a downstream effector of Notch signaling in regulating NSCs. Previously, we have reported that miR-342-5p functions as a direct downstream effector of Notch signaling regulating the differentiation of NSCs into INPs, and the commitment into astrocytes (Gao et al., 2017). Therefore, Notch signaling maintains the stemness of NSCs at different processes by modulating miR-582-5p and miR-342-5p, respectively.

Although miR-582-5p promotes the stemness and proliferation of NS/PCs *in vitro*, there was an increase of Ki67^+^ cells in the SVZ of miR-582 KO mice compared with that of control. In miR-582-5p KO mice, the number of both NSCs and TAPs decreased, and neuroblasts increased, suggesting accelerated differentiation into neuroblast, and exhaustion of NSCs and TAPs. Therefore, the increase of Ki67^+^ cells in the SVZ of KO mice might be related to increased proliferation of neuroblasts or niche cells.

In our study, the gain of function of miR-582-5p could promote the formation of neurospheres and proliferation of NS/PCs. This suggests that miR-582-5p ago-miRs could be used to promote the expansion of NSCs *in vitro*. In human, miR582-5p and miR-582-3p have been shown to regulate cancer cell proliferation and cancer stem cell maintenance, respectively (Maeno et al., 2014; Fang et al., 2015), indicating that their functions might be evolutionarily conserved in human. However, whether miR-582-5p could promote the expansion of human NSCs need further experimental confirmation. More importantly, we have also observed reduced NSCs and enhanced neurogenesis in the SVZ of miR-582-5p deficient mice. Since activated neurogenesis in adult brain has been related to injury repair, learning and memory, and anxiety (Goncalves et al., 2016), miR-582-5p might provide a new target to modulate adult neurogenesis and the behavior consequences *in vivo*. On the other hand, although miR-582-5p promoted the formation of neurospheres *in vitro*, its accurate function and mechanism on the self-renewal of NSCs needs further study. Sequential neurosphere formation and the apoptosis of NSC should be thoroughly analyzed to acquire solid evidence of self-renewal ability and to exclude defects in NSC survival after miR-582-5p overexpression.

In term of upstream regulation, although Notch signaling regulates the expression of miR-582-5p, our data suggest that it does not directly regulate the expression of its host gene Pde4d. The genes encoding miRNAs are frequently embedded in the intron or exon of host genes, and the expression of miRNAs is co-regulated with host genes under the same set of promoter-enhancer elements. These property makes it possible that miRNAs and their host genes could be coordinately regulated under certain physiological or pathological status to maintain homeostasis. For example, miR-33 is an intronic miRNA located within the gene encoding sterol-regulatory element-binding factor-2 (SREBF-2). It has been demonstrated that miRNA-33 and the SREBP host gene cooperate to control cholesterol homeostasis by regulating the expression of genes involved in cellular cholesterol transportation (Najafi-Shoushtari et al., 2010; Rayner et al., 2010). This may be an evolution-associated mechanism controlling gene expression pattern and the downstream biological functions. However, in some other cases, though the miRNA genes are within the introns of their host genes, the miRNA genes are transcribed in a host gene-independent manner because of the complexity of gene expression regulation mechanisms. As we have reported here, although Notch signaling regulates the expression of miR-582-5p, it does not directly regulate the host gene Pde4d. This adjacent but independent mode of transcriptional regulation further increases the diversity of gene expression regulation mechanisms. The precise mechanisms by which Notch signaling modulates miR-582-5p need further analysis in the future.

Finally, the downstream effector FAM19A1 has been reported to act as a ligand for GPCR1, and to regulate NSC proliferation and differentiation through Rho-associated protein kinase (ROCK)/ERK1/2, and ROCK/signal transducer and activator of transcription 3 signaling pathways (Zheng et al., 2018). However, whether Notch-miR-582-5p-FAM19A1 axis regulate NSCs through the same mechanism requires further study.

In summary, by overexpression and downregulation of miR-582-5p in NC/PCs *in vitro*, we have found that miR-582-5p could participate in maintaining the stemness and proliferation of NSCs. Further analysis on miR-582-5p knockout mice has shown that miR-582-5p deficiency reduces NSCs and enhances neurogenesis in the SVZ, indicating that miR-582-5p also maintains NSC stemness *in vivo*. To access the underlying molecular mechanism, we have also carried out RNA-seq followed by molecular biological analyses, which reveal that miR-582-5p promotes stemness and proliferation of NSCs by targeting secretory protein FAM19A1. Taken together, our work has uncovered novel miRNA-mediated epigenetic mechanism regulating NSC maintenance.

## Data Availability Statement

The datasets presented in this study can be found in online repositories. The names of the repository/repositories and accession number(s) can be found below: https://www.ncbi.nlm.nih.gov/sra/?term=PRJNA806980.

## Ethics Statement

The animal study was reviewed and approved by the Animal Experiment Administration Committee of the Fourth Military Medical University.

## Author Contributions

Y-FZ and X-XL performed experiments and collected the data. X-LC, C-CJ, X-YG, and DG assisted with experiments and data collection. HH, FY, and M-HZ designed the experiments and prepared the manuscript. All authors contributed to the article and approved the submitted version.

## Conflict of Interest

The authors declare that the research was conducted in the absence of any commercial or financial relationships that could be construed as a potential conflict of interest.

## Publisher’s Note

All claims expressed in this article are solely those of the authors and do not necessarily represent those of their affiliated organizations, or those of the publisher, the editors and the reviewers. Any product that may be evaluated in this article, or claim that may be made by its manufacturer, is not guaranteed or endorsed by the publisher.

## Footnotes

1
http://www.omicshare.com/tools
