# Supplementary material for: MicroRNA-582-5p Contributes to the Maintenance of Neural Stem Cells Through Inhibiting Secretory Protein FAM19A1
Source: Front Cell Neurosci. 2022 May 24;16:866020. doi: 10.3389/fncel.2022.866020 (PMC9171424; doi:10.3389/fncel.2022.866020)
Supplement: Supplementary file 1 [file Data_Sheet_1.pdf]

Supplemental Information

Supplemental figure 1

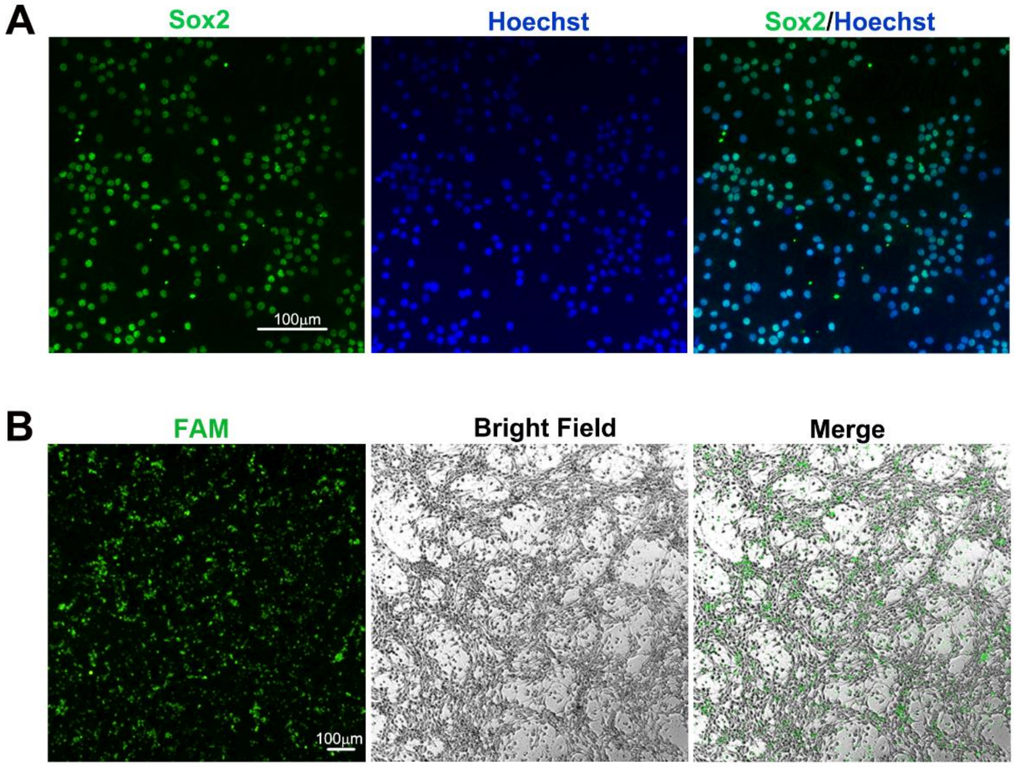

Sup Figure 1: (A) Identification of NS/PCs by Sox2 immunostaining. Scale bar = 100 μm. (B) Transfection efficiency of NS/PCs was detected by transfection of scrambled control oligonucleotides labeled with FAM. Scale bar = 100 μm.

Supplemental figure 2

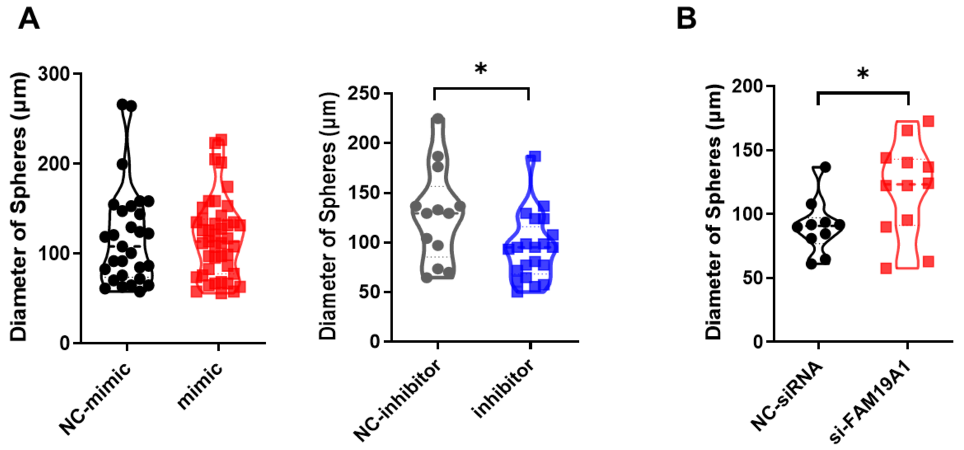

Sup Figure 2: The diameter of spheres. (A) The diameter of spheres in the NC-mimic and mimic groups, and in NC-inhibitor and inhibitor groups, n=3, * $p < 0.05$. (B) The diameter of spheres in the NC-siRNA group and si-FAM19A1 group, n=3, * $p < 0.05$.

Supplemental figure 3

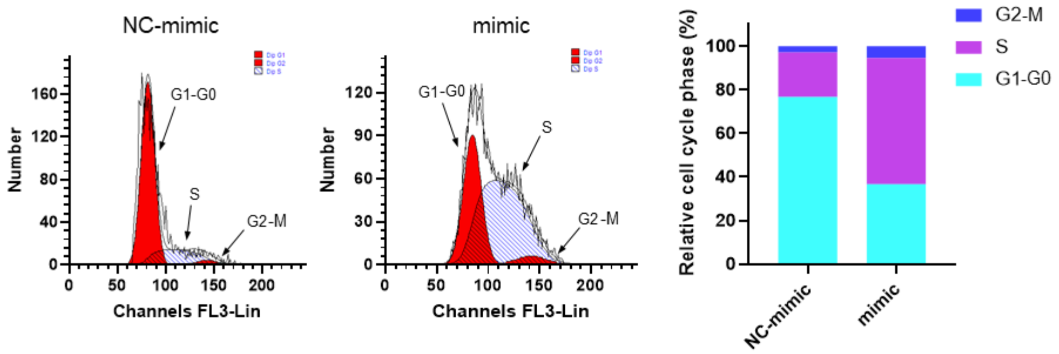

Sup Figure 3: Cell cycle analysis by flow cytometry showed the results of NC-mimic group and mimic group, and the ratio of G1-G0, S and G2-M phases in these two groups.

Supplemental figure 4

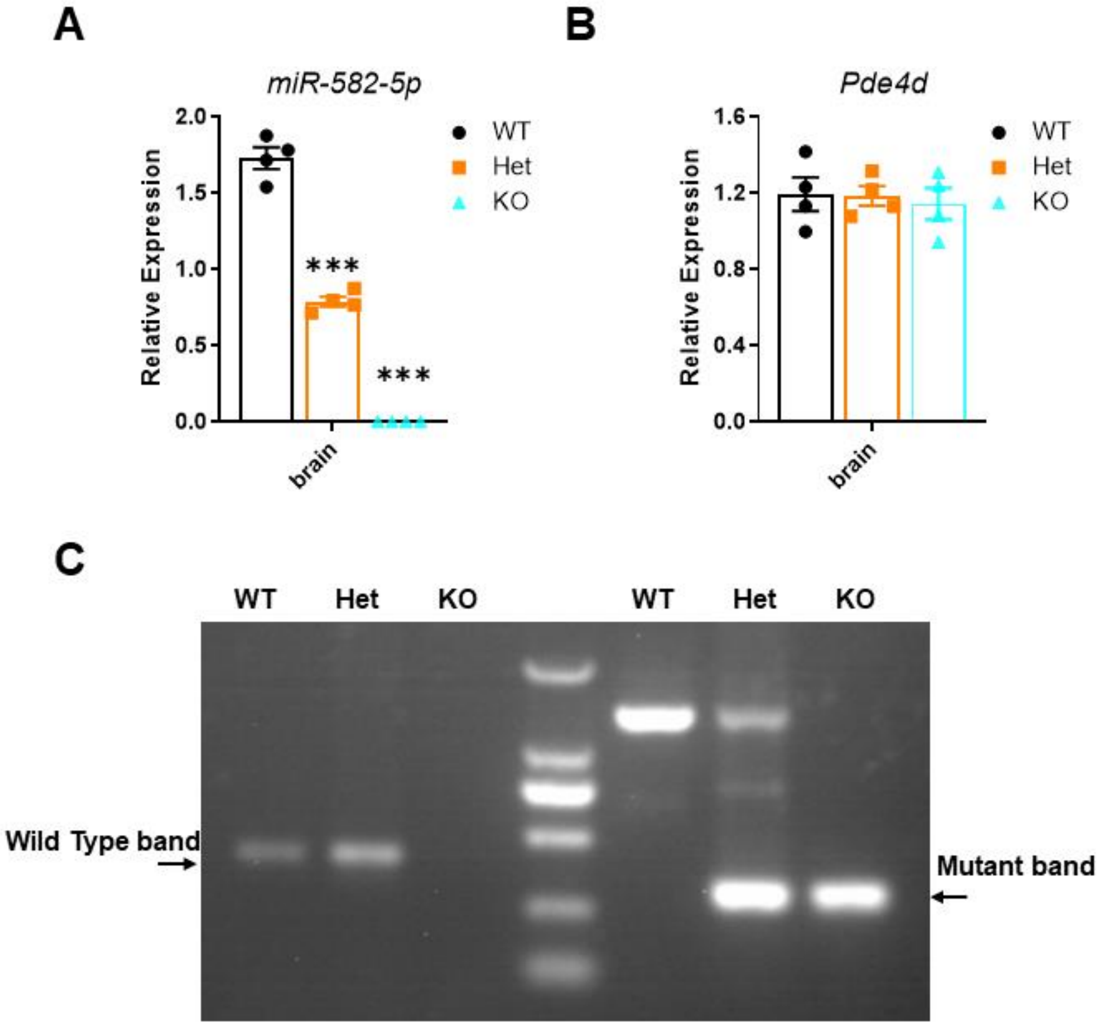

Sup Figure 4: The detection of miR-582-5p and Pde4d expression and genotyping of WT, Het and miR-582 KO mice. (A) The expression of miR-582-5p was declined in Het mice and absent in KO mice, n=4, *** $p < 0.001$. (B) There was no difference in the expression of host gene Pde4d among the three groups, n=4. (C) Gel electrophoresis results for genotyping, with wild type band (414 bp), and mutant band (298 bp).

Supplemental figure 5

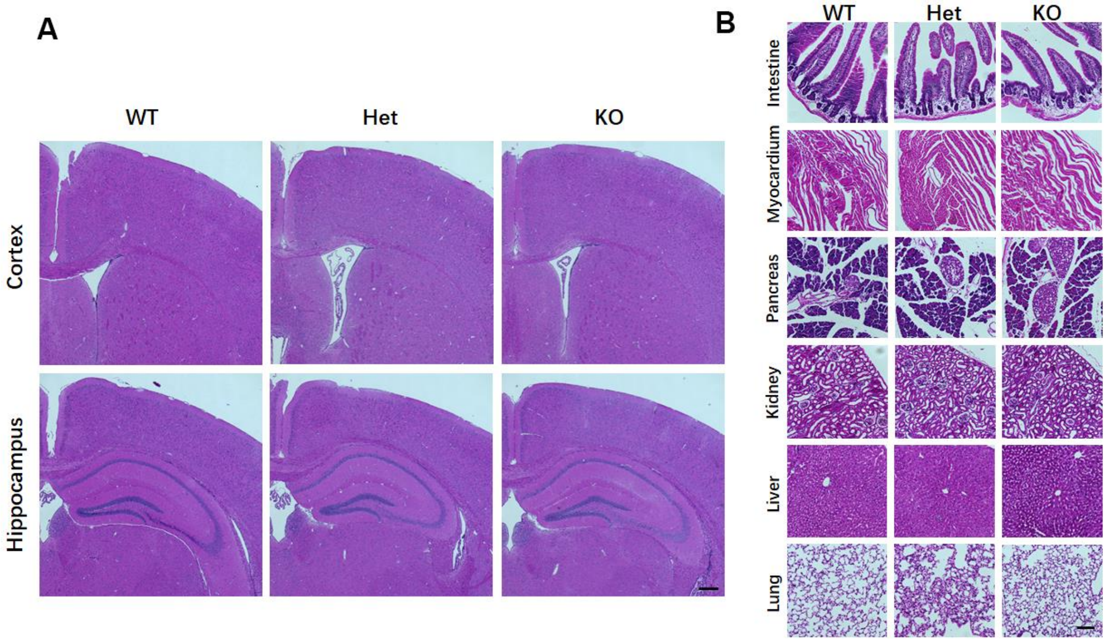

Sup Figure 5: Hematoxylin-eosin (H&E) staining of brain and organ sections from WT, HET and miR-582 KO mice were comparable. A. H&E staining of cortex and hippocampus. B. H&E staining of various organ sections. Scale bar = 100 μm in A and B.
